# Commentary: Cubital Tunnel Release Under Local and Regional Anesthesia: A Scoping Review

**DOI:** 10.1177/22925503261477030

**Published:** 2026-08-10

**Authors:** Donald H. Lalonde

**Affiliations:** 1 Division of Plastic Surgery, 3688 Dalhousie University, Saint John, Canada

The authors have written an excellent scoping review that compares the literature complication rates of cubital tunnel release with different anesthesia techniques.^1^ The authors conclude from their literature search that the complication rates after regional (2.3%) and local anesthetic (2.9%) techniques are similar to the complication rates of cubital tunnel release after general anesthesia (2.5%). However, a serious shortcoming of the literature they reviewed is that most of the papers do not even consider nausea and vomiting as a complication.

A 2026 anesthesia literature paper compared modern technique remimazolam total intravenous general anesthesia to inhalational sevoflurane-based balanced general anesthesia and found a nausea and vomiting rate of 20% (123/625 patients).^2^ There is no question that the anesthesia paper 20% nausea and vomiting rate is much higher than the cubital tunnel scoping review paper report of a 2.5% general anesthesia complication rate.

Nausea and vomiting are not associated with (Wide Awake Local Anesthesia No Tourniquet (WALANT) surgery because there is no sedation.^3^ Nausea and vomiting are so common with sedation and general anesthesia that this is not even considered a complication by many. It is “expected.” Let's be honest. Nausea and vomiting not only make us all feel terrible, but it causes post-operative bleeding and hematoma, which are then considered “surgical” complications when the inciting cause is the sedation and not the surgery.^4^

This discussion is a plea for those who write surgical complication papers to start including nausea and vomiting as part of the complication rate when sedation is used as part of the surgery. Even WALANT papers should report the usual 0% nausea and vomiting complication rate to make the point that it rarely, if ever, happens with no sedation.
